# Editorial: Robotic surgery: Human learning, simulation and training on surgical education

**DOI:** 10.3389/fsurg.2022.1061691

**Published:** 2022-11-11

**Authors:** Ka-Chun Siu, Francisco Schlottmann

**Affiliations:** ^1^ Department of Health and Rehabilitation Sciences, University of Nebraska Medical Center, Omaha, NE United States; ^2^ Department of Surgery, Hospital Alemán of Buenos Aires, Buenos Aires, Argentina; ^3^ Department of Surgery, University of Illinois at Chicago, Chicago, IL United States

**Keywords:** medical education, surgical training, minimally invasive surgery, simulation technology, outcome assessment

**Editorial on the Research Topic** Robotic surgery: Human learning, simulation and training on surgical education By Siu K-C and Schlottmann F. Front. Surg. (2022) **9**: 1061691. doi: 10.3389/fsurg.2022.1061691

In the last two decades, the innovations in surgical training, the advancement of robotic technology, and simulation have pretended potential solutions to provide a safe, standardized, and cost-effective training environment for surgical trainees (1, 2). However, one of main challenges of surgical education is providing life-like training environments to develop skills readiness, skill maintenance, and skill retention (3–5). It is essential to constantly evaluate human learning and ergonomics to assess the use of robotic technology and simulation on surgical education and practice.

Providing adequate and effective training for future surgeons while ensuring safety and feasibility of the robotic approach remains critical. In this research topic, several studies are presented to showcase how robotic surgery can be adopted and safely performed in unique procedures with positive clinical outcome and how to better assess robotic surgical outcome longitudinally.

The use of the robotic platform in surgery has been expanded in many regions from developing to underdeveloping countries. Erdemir and Rasa reported their initial experience in a Turkish regional hospital on how to use surgical robot to perform adrenalectomy. They assessed their robotic adrenalectomy experience (30 cases) and outlined the factors that have a significant impact on surgical outcome, and concluded that robotic adrenalectomy is effective and safe for adrenal gland pathologies.

Whether a new procedure and/or approach is safe to perform is the first and critical step for all surgeries including robotic surgery. Wang et al. reported their first experience in robotic hepatectomy with middle hepatic vein reconstruction using expanded polytetrafluoroethylene graft for a patient with hepatic adenoma. The graft was patent after the robotic procedure and patient's liver function was well recovered at the follow-up of 3 months after surgery. This case study demonstrates that robotic hepatectomy with middle hepatic vein reconstruction is a safe alternative for patients with complex liver tumors.

Xie et al. also evaluated the safety and postoperative outcomes of revisional bariatric surgeries using a minimally invasive approach at an accredited high-volume medical center. Using the 2015–2017 Metabolic and Bariatric Surgery Accreditation and quality improvement program database, the authors found that minimally invasive revisional bariatric surgery can be safely performed in different patient populations with low conversion and less complication rates, and improved weight loss.

Besides safety, selecting an appropriate surgical technique to perform robotic surgery is another important question to explore. Zhang et al. investigated the use of the double purse-string suture technique for circular-stapled anastomosis during robotic Ivor Lewis esophagectomy. They evaluated 10 robotic cases using this technique and no anastomosis-related complications were observed. Thus, this robotic anastomotic technique is a promising alternative that should be further explored in esophageal cancer patients undergoing Ivor Lewis esophagectomy.

In order to better assess clinical outcome, the literature has suggested different methods for consideration. Huang et al. used the cumulative sum (CUSUM) method to analyze the learning curve of robotic hemicolectomy for colon cancer. This retrospective study examined 76 cases at a single center and showed two stages of learning from initial learning stage to proficiency phase. It seems that learning to perform hemicolectomy reaches the plateau after 27 cases, and the operation time and intraoperative blood loss are reduced as more cases are performed. A retrospective study by Wang et al. also applied the CUSUM method to examine the learning curve for robotic single-anastomosis duodenal-ileal bypass with sleeve gastrectomy. They studied 102 consecutive patients and concluded that robotic single-anastomosis duodenal-ileal bypass with sleeve gastrectomy is a feasible, safe, and reproducible surgical technique with a learning curve of 58 cases.

The growing body of literature including clinical trials and systematic reviews has supported the effective use of robotic-assisted surgery in hospitals (6–8). Lelpo et al. developed a comprehensive protocol for a perspective, multicentric, observational study. This Spanish national study will explore robotic and laparoscopic surgical procedures. Seven surgical operations and several patient populations will be included. This important study will shed light on the economic impact of robotic surgery on the healthcare system compared with laparoscopic surgery.

Overall, this research topic highlights several successful and safe procedures that could be performed robotically. Learning robotic surgery to obtain positive clinical outcome requires practice and training. The CUSUM method is commonly used to study human learning progress during robotic surgical procedure. Although successful examples are presented, they are still at the initial feasibility phase and need larger clinical studies or even randomized controlled trials to establish the stage for clinical recommendations. Therefore, future studies should explore how robotic surgery can be safely trained, embraced, and performed.
